# Nanopore direct RNA sequencing for RNA modification analysis: workflow assessment and computational tool benchmarking

**DOI:** 10.1007/s44307-025-00093-5

**Published:** 2026-03-10

**Authors:** Zhixing Wu, Jiayi Li, Rong Xia, Jiayin Dai, Jionglong Su, Jia Meng, Yuxin Zhang

**Affiliations:** 1 Department of Biosciences and Bioinformatics, Center for Intelligent RNA Therapeutics, Suzhou Key Laboratory of Cancer Biology and Chronic Disease, School of Science, Xi’an Jiaotong-Liverpool University, Suzhou, Jiangsu 215123 China; 2 School of AI and Advanced Computing, XJTLU Entrepreneur College, Xi’an Jiaotong-Liverpool University, Suzhou, China; 3 Department of Computer Science, University of Liverpool, Liverpool, L7 8TX UK; 4 Institute of Systems, Molecular and Integrative Biology, University of Liverpool, Liverpool, L69 7BE UK; 5 Key Laboratory of Ministry of Education for Gastrointestinal Cancer, School of Basic Medical Sciences, Fujian Medical University, Fuzhou, Fujian 350004 China; 6 State Key Laboratory of Epigenetic Regulation and Intervention, Institute of Biophysics, Chinese Academy of Sciences, Beijing, 100101 China

**Keywords:** Nanopore sequencing, Analytical pipelines, RNA modification detection, Computational tools, Machine learning, Deep learning, Base calling, Benchmark analysis

## Abstract

Recent advancements in sequencing technologies have transformed the characterization of genomic and transcriptomic complexity. In this review, we present a comprehensive overview of Oxford Nanopore Technologies (ONT), emphasizing its unique capability for real-time, long-read, and direct RNA sequencing. We begin by outlining the core ONT analytical workflow—base calling, alignment, re-squiggling, and quality control—and summarize the major computational tools applied at each stage. Then extensive illustrations of various RNA modification detection techniques are provided, spanning from statistical models, machine learning and deep learning frameworks to advanced strategies incorporating large language models. To assess methodological performance, additional benchmark analyses of m6A and pseudouridine (Ψ) are carried out across two publicly available datasets. These results demonstrate substantial variability across different tools, underscoring the inherent difficulties in reliably detecting modifications from ONT signals. We further examine the biological roles of key RNA modifications and contrast ONT-based approaches with conventional detection technologies. Finally, we discuss persistent limitations such as sequencing error rates, data and computational demands, and the complexity of multi-modification inference, and further propose future directions aimed at improving accuracy, robustness, and biological interpretability in ONT-based epitranscriptomic research.

**Supplementary Information:**

The online version contains supplementary material available at 10.1007/s44307-025-00093-5.

## Background and sequencing generations

The field of sequencing has significantly evolved over the past decades, driven by advancements in biochemistry, molecular biology, and computational analysis (Deamer, et al. 2016; Shendure, et al. 2017; McGinn and Gut 2013). The progressions through three distinct generations of sequencing technologies have highlighted efforts to enhance throughput, read length, accuracy, and affordability, which are essential for addressing complex biological questions (Estrada-Rivadeneyra 2017; Deamer, et al. 2016; McGinn and Gut 2013).

**Fig. 1:**
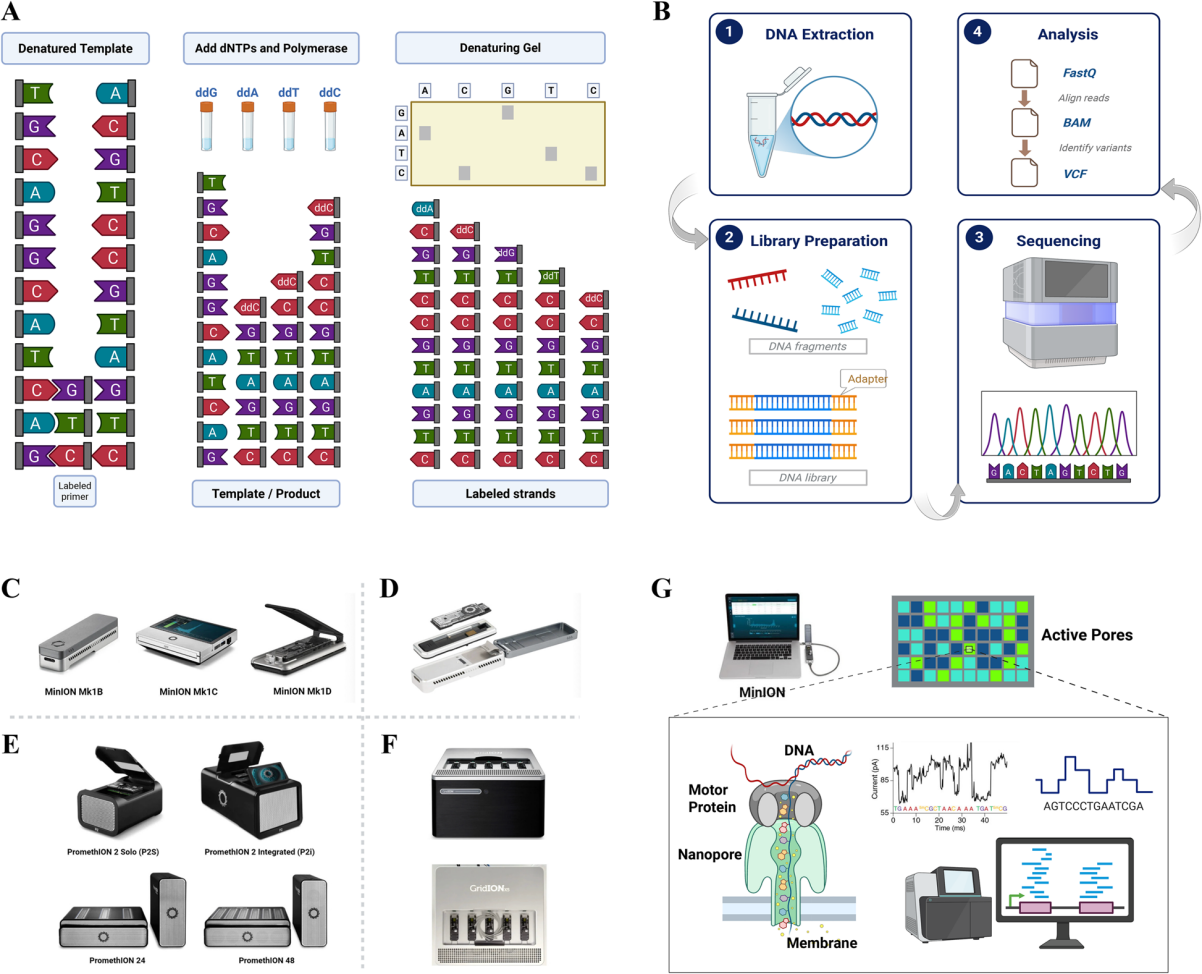
Workflow summaries of Sequencing methods with key procedures and devices (Wang, et al. 2021; Kchouk, et al. 2017; Tamang 2024; Sagar 2019). **A** Overall Diagram of Sanger First-generation Sequencing Method. **B** Four fundamental steps for the NGS framework comprising Extraction, Preparation, Sequencing, and Analysis. **C-F** Devices introduced by Oxford Nanopore Technology (ONT): MinION Products (**C**), Flongle adapter (**D**), PromethION (**E**), and GridION products (**F**). **G** Simplified procedures and elements of a representative TGS technology of ONT Nanopore Sequencing. Note: This Figure has been created in BioRender https://BioRender.com

First-generation Sequencing, pioneered by Sanger et al. in the 1970s, mainly relies on chain termination reactions and electrophoretic separation to determine nucleotide sequences (Estrada-Rivadeneyra 2017) (Fig. 1A). While this method has set the foundation for modern genomics due to its precision and reproducibility, it is limited by relatively short read lengths (typically < 1,000 bases) and low throughput, rendering it impractical for large-scale projects such as whole-genome sequencing (Estrada-Rivadeneyra 2017; Deamer, et al. 2016; McGinn and Gut 2013).

Second-generation Sequencing (SGS) or Next-generation Sequencing (NGS) has introduced platforms such as Illumina and Roche 454, which rely on cyclic reversible termination and massively parallel sequencing (McGinn and Gut 2013; Deamer, et al. 2016; van Dijk, et al. 2014). These methods significantly increase throughput with reduced costs, facilitating extensive genomics and transcriptomics analyses (van Dijk, et al. 2014; Kchouk, et al. 2017; McCombie, et al. 2018; Sagar 2019) (Fig. 1B). Despite these advances, NGS technologies are constrained by their short read lengths (~ 100–300 bp), hindering resolution of complex genomic regions and repetitive elements (McCombie, et al. 2018; Xuan, et al. 2013; Rodriguez and Krishnan 2023). Additionally, the reliance on amplification methods might introduce biases, potentially obscuring further epigenetic modifications (Shendure, et al. 2017; Kchouk, et al. 2017; Amarasinghe, et al. 2020).

Third-generation Sequencing (TGS) technologies, notably Pacific Biosciences (PacBio) and Oxford Nanopore Technologies (ONT), have addressed several limitations by enabling single-molecule, long-read sequencing without amplification (Cuber, et al. 2023; McGinn and Gut 2013; Lin, et al. 2021; Amarasinghe, et al. 2020; PacBio 2020; Zhang, et al. 2024a, b). PacBio’s Single Molecule Real-Time (SMRT) sequencing could generate long, high-fidelity reads by employing zero-mode waveguides and fluorescent base detection (Rhoads and Au 2015; Cuber, et al. 2023). ONT’s nanopore sequencing detects ionic current changes as DNA or RNA molecules translocate through nanopores (Cuber, et al. 2023; Hrdlickova, et al. 2016; Deamer, et al. 2016; Seki, et al. 2018), enabling real-time, label-free sequencing and direct detection of native chemical modifications such as 5-methylcytosine (5mC), N6-methyladenosine (m6A), and pseudouridine (Ψ) (Seki, et al. 2018; Cuber, et al. 2023; Brown and Clarke 2016; Tamang 2025).

Nanopore Sequencing comprises three major categories: direct DNA sequencing (i.e., native genomic DNA preserving modifications) (Dorey and Howorka 2024; Shendure, et al. 2017; Tyagi and Bhide 2020; Heather and Chain 2016), direct RNA sequencing (i.e., native RNA molecules and transcript structure detection) (Cuber, et al. 2023; Amarasinghe, et al. 2020; Hrdlickova, et al. 2016), and cDNA sequencing (i.e., reverse transcription-based, stable reads but loss of native epitranscriptomic information) (Bashiardes and Lovett 2001; Hu, et al. 2021). These strategies could support diverse applications from structural variant analysis, single-molecule transcriptomics and methylome profiling, to further diagnostics and genome assembly (Seki, et al. 2018; Lin, et al. 2021; Ying, et al. 2022; Lu, et al. 2016; Sheka, et al. 2021).

## ONT nanopore sequencing: analytical workflows and practical tools

In contrast to first- and second-generation sequencing technologies, which typically produce short reads ranging from 50 to 500 bp fragments, TGS offers long-read capabilities, routinely generating reads spanning tens of kilobases (Zhang, et al. 2024a, b; Tamang 2025). Among TGS technologies, Oxford Nanopore Technologies (ONT) nanopore sequencing stands out as a transformative advancement, enabling real-time, direct sequencing of long nucleic acid molecules. Over the past decade, ONT has released a range of devices with improved performance and flexibility (Lu, et al. 2016; Brown and Clarke 2016). Notable instruments include the portable MinION (2015, Fig. 1C), the Flongle adapter (2019, Fig. 1D) for smaller-scale experiments (Tamang 2025), the high-throughput PromethION (2018, Fig. 1E), and the GridION (2017, Fig. 1F) capable of running five MinION flow cells in parallel.

**Fig. 2:**
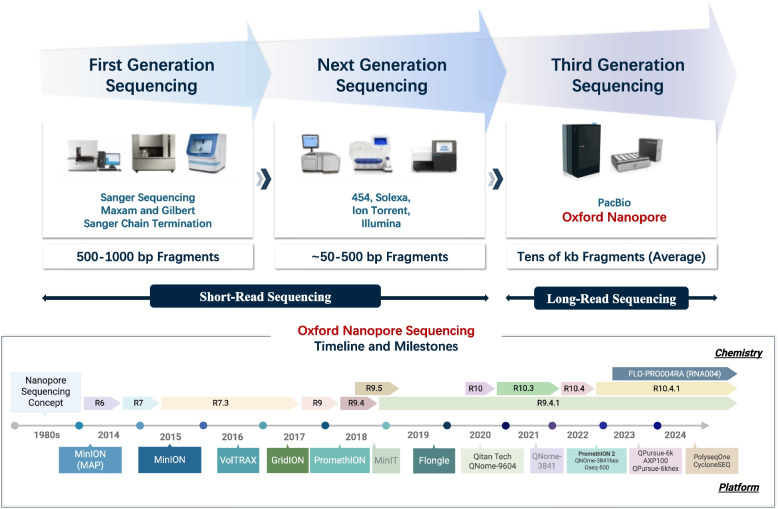
Brief Comparisons of three Sequencing phases with representative devices and features, as well as Timeline of Oxford Nanopore with diversified Chemistry and Platforms (PacBio 2020; Zhang, et al. 2024a, b; nanoporetech 2025a, b, c, d, e; Wang, et al. 2021). Note: This Figure has been created in BioRender https://BioRender.com

Concurrently, ONT’s sequencing chemistry (Fig. 1G) has undergone significant iterations. The R9 chemistry emerged around 2017, and the version of R9.4.1 has been widely adopted since 2019. The R10 series, introduced near 2020, has brought improvements in accuracy and resolution, with subsequent versions such as R10.3 and R10.4 further enhancing performance (Zhang, et al. 2024a, b; Wang, et al. 2021). Most recently, FLO-PRO004RA (RNA004) was released for advanced sequencing applications (Zhang, et al. 2024a, b). A timeline summarizing ONT chemistry and platform evolution is provided in Fig. 2.

In terms of the primary analytical pipeline for nanopore sequencing, it comprises several essential stages: base calling, alignment, signal re-squiggling, and quality control. Each of these components has benefited from the integration of computational frameworks and algorithmic innovations, substantially improving data accuracy and processing efficiency. This section will provide a comprehensive overview of the core stages, detailing their function, representative tools, underlying methodologies, and recent investigations.

### Base calling

**Fig. 3:**
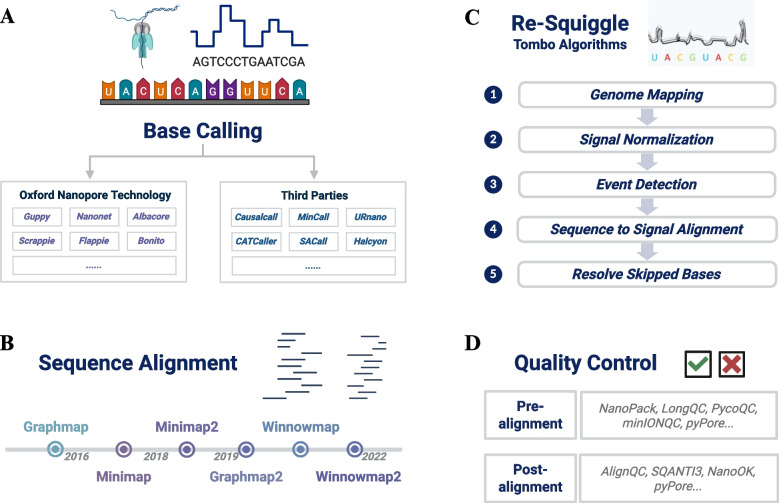
Pipelines and Key Procedures regarding Oxford Nanopore Technologies (ONT) Nanopore Sequencing primary analysis (Sahlin, et al. 2023; OxfordNanoporeTechnologies 2017a,b, n.d.a, b, 2021a, b; Wang, et al. 2021). **A** Initial step of Base Calling with examples of tools from ONT and third parties. **B** Timeline of some well-known alignment tools that aim at aligning sequences against the reference databases. **C** The new Re-squiggle making assignment from squiggle to reference sequence, with the illustration of five steps for the Tombo algorithms. **D** Various Quality Control instruments utilized in both the pre-alignment and post-alignment phases. Note: This Figure has been created in BioRender https://BioRender.com

Base calling refers to the computational conversion of raw ionic current signals into nucleotide sequences when nucleic acid polymers traverse the nanopores (OxfordNanoporeTechnologies n.d.a, b, 2021a, b; nanoporetech 2025a, b, c, d, e). This process is inherently influenced by the sequence context due to the overlapping K-mer signals (typically 5-mers) characteristic of ONT platforms (nanoporetech 2025a, b, c, d, e; Wang, et al. 2021). Contemporary base calling tools can be grouped into two categories, one with ONT-provided software (e.g., *Guppy*, *Nanonet*, *Bonito, Albacore, Scrappie)*, while the other belongs to Third-Party tools (e.g., *Causalcall, SACall, MinCall)* (Fig. 3A).

Early base callers employed Hidden Markov Models (HMM) to model signal variability, but their performance was limited in handling signal heterogeneity (Wang, et al. 2021). In late 2017, ONT’s flagship neural base caller, Guppy (nanoporetech 2025a, b, c, d, e; timkahlke n.d.), introduced Long Short-Term Memory (LSTM) based Recurrent Neural Networks (RNN) to model temporal dependencies in the ionic trace, yielding accuracy gains over HMM (OxfordNanoporeTechnologies 2021a, b; Zhang, et al. 2020; nanoporetech 2025a, b, c, d, e). Subsequently, ONT has introduced several computational approaches like *Nanonet, Albacore, Scrappie* (nanoporetech 2019)*, and Flappie* (nanoporetech 2020a, b), while *Guppy* still remained relatively preeminent with well-performed accuracy metrics and fast speed due to the GPU acceleration (Wick, et al. 2019; nanoporetech 2025a, b, c, d, e).

*Bonito* (nanoporetech 2024), an open-source ONT base caller employing Convolutional Neural Networks (CNN) for rapid inference, demonstrated > 1% accuracy improvements compared to the previous *Guppy* (Wang, et al. 2021; nanoporetech 2025a, b, c, d, e; OxfordNanoporeTechnologies n.d.a, b; Loose, et al. 2016; Wick, et al. 2019; Xu, et al. 2021). The optimized variant, *Fast-Bonito*, further accelerated inference via lightweight CNN architectures and network pruning, achieving speeds up to 53.8% faster than *Bonito* on NVIDIA V100 GPUs (Wick, et al. 2019; Pagès-Gallego and de Ridder 2023; Xu, et al. 2021). Similarly, other tools like *PoreOver* made further improvements towards *Bonito* by showing additional error reduction (Xu, et al. 2021; Silvestre-Ryan and Holmes 2021).

Third-party base callers have also advanced the state of the art. Several models such as *Mincall* (Miculinić, et al. 2019; He, et al. 2016) and *Causalcall* (Zeng, et al. 2020) used deep CNN and causal-dilated CNN respectively. *SACall* (Huang, et al. 2020) and *CATCaller* adopt transformer encoders (Wu, et al. 2020), *URNano* (Zhang, et al. 2020) applied a convolutional U-net with RNNs, while *Halcyon* (Konishi, et al. 2020) employed the method of Attention to construct a sequence-to-sequence framework (Pagès-Gallego and de Ridder 2023). According to the comparisons of these seven base calling models, *Bonito*’s design outperforms the remaining six of *MinCall*, *CausalCall, URNano, SACall, CATCaller*, *Halcyon* with its superior architecture (Pagès-Gallego and de Ridder 2023). Detailed architectures, including the convolution, encoder, and decoder components of these tools, have been summarized in Supplementary Table 1.

More recently, another milestone of *Dorado* (nanoporetech 2022) has been introduced, which could represent the new era of base calling evolution. It has relied on Deep Neural Networks (DNN) and advanced layers to ensure accuracy. *Dorado* was once proven to achieve around 99.5% raw read accuracy, and even with higher gains on upgraded flow cells and model versions (Pagès-Gallego and de Ridder 2023; Wick, et al. 2019; OxfordNanoporeTechnologies n.d.a, b, 2021a, b).

Together, these advancements have substantially improved ONT sequencing accuracy, making it a competitive platform that rivals short-read sequencing technologies in terms of throughput, precision, and functional versatility.

### Alignment

Accurate alignment of long, error-prone nanopore reads is essential for numerous downstream applications, including variant calling, structural variant discovery, genome assembly, and epigenetic profiling (OxfordNanoporeTechnologies 2021a, b; Wang, et al. 2021; Sahlin, et al. 2023). Given the unique characteristics of nanopore reads—namely their variable lengths, high error rates (particularly with insertions and deletions), and the presence of modified bases—the development of robust alignment algorithms has been a priority in the field (Wang, et al. 2021; OxfordNanoporeTechnologies n.d.a, b; Seki, et al. 2018; Sahlin, et al. 2023). Milestones of several alignment tools were labelled in Fig. 3B.

*GraphMap* (isovic 2016) is among the first nanopore-specific aligners. It has introduced a gapped q-gram seeding strategy allowing for fast search, which could improve sensitivity to indels and mismatches, especially in noisy regions. Evaluation based on MinION data against short and long read mappers could represent the improved mapping sensitivity by 10–80% and > 95%, respectively, of bases mapping results for the *GraphMap* tool (Sahlin, et al. 2023).

Subsequent innovations include *Minimap* emerged around 2018, as well as the introduction of *Minimap2* (Li 2022; Sahlin and Mäkinen 2021; Li 2018) which has become the widely applied aligner for long-read sequencing due to its impressive speed and versatility (Li 2018). It has superseded earlier mappers by using split-read alignment and delivering 3–4 times as fast as mainstream short-read mappers at the comparable accuracy levels (Li 2018). Additionally, *Minimap2* could support both DNA and long mRNA alignments, making it suitable for genome assembly and transcriptome analysis. More tools have also been refined, such as the tool of *uLTRA*, which was introduced in 2021 and worked as a wrapper around *Minimap2* to align reads outside annotated regions (Sahlin and Mäkinen 2021; Sahlin, et al. 2023). Further adoption of newly developed algorithms has contributed to the *Graphmap2* (lbcb 2020; OxfordNanoporeTechnologies 2019), which has achieved better performance of higher mappability as well as more recognized exons and their ends than previous *Minimap2* and *Graphmap* under the same simulated or real datasets (Sahlin, et al. 2023; OxfordNanoporeTechnologies 2019).

In 2018, Sedlazeck et al. introduced a long-read alignment tool called *NGMLR* (philres 2018) and a structural variant identification method called *Sniffles* (Sedlazeck 2025). Such approaches would contribute to the cost reduction for long reads due to the automatic filtering of false events and operations on low-coverage data, becoming highly useful under clinical or human health-related settings. *Winnowmap2*, released in 2022, has utilized minimal confidently alignable substrings and showed more tolerance of structural variation and more sensitivity to paralog-specific variants within repeats (marbl., 2021; Jain, et al. 2022). Based on performance evaluation, the lowest false-negative and false-positive rates were observed in *Winnowmap2* with merely (1.89%, 1.89%), in comparison with *Minimap2* (39.62%, 5.88%) and *NGMLR* (56.60%, 36.11%), respectively (Jain, et al. 2022).

These aligners have highlighted the trade-offs among speed, sensitivity, and signal awareness. *Minimap2* remains a robust and general-purpose aligner, balancing performance and resource usage across applications (Li 2018). *GraphMap2* serves as a sensitive aligner with a large improvement in mappability compared to previous *GraphMap* and *MiniMap2* (Li 2018; OxfordNanoporeTechnologies 2019). Further tools like *NGMLR* and *Sniffles* deal with structural variants (philres 2018; Sedlazeck 2022), while *Winnowmap2* fills a critical aspect in terms of repetitive reference sequences with extraordinary performance (Jain, et al. 2022). Recent findings in the year 2025 towards *Uncalled4* have also made great breakthroughs by providing a fast and accurate method for signal alignment, which could improve the DNA and RNA modification processes accordingly (Kovaka, et al. 2025).

### Re-squiggle

This re-squiggling step involves aligning raw signals to reference sequences, promoting further refinement of base-level annotations and detection of modifications. For instance, the tool of *Nanopolish* employs HMM and statistical comparison between methylated and unmethylated models to detect 5mC and further extends the method to human DNA (nanoporetech 2020a, b; Simpson, et al. 2017). Moreover, tools like *Tombo* (OxfordNanoporeTechnologies 2017a, b; nanoporetech 2020a, b) re-map raw electrical current signals to genome positions, allowing for the identification of base modifications via statistical deviations from expected current levels (nanoporetech 2020a, b). A similar tool called *Remora* could also conduct the analysis and is capable of making predictions about methylation bases with high performance (OxfordNanoporeTechnologies 2021a, b; nanoporetech 2024a, b).

Specifically, *Tombo* performs this re-squiggling operation via a five-stage pipeline, comprising mapping, normalization, event detection, sequence-to-signal assignment, and base-skipping resolution (OxfordNanoporeTechnologies 2017a, 2017b) (Fig. 3C). The initial step of Genome Mapping aligns previous nucleotide sequences to a reference genome to establish a foundational coordinate system, which could be achieved by tools like *Minimap2*. The second Signal Normalization procedure normalizes raw ionic current signals to mitigate experimental variability and systematic biases, and *Tombo* could further utilize change-point detection algorithms to identify events in the third phase of Event Detection. Then, *Tombo* employs dynamic programming techniques for optimized Sequence-to-Signal Assignment and applies special routines to handle under-segmentation or noisy regions in the final Resolving Skipping Bases part. While this represents *Tombo*’s specific implementation, most re-squiggling tools adopt a similar conceptual workflow, comprising these core stages despite some differences in algorithmic details or software designs.

### Quality control

Robust quality control (QC) is critical to ensure that the generated sequencing data could meet accuracy and completeness criteria necessary for reliable downstream analyses. Examples of both pre-alignment and post-alignment QC tools could be found in Fig. 3D.

Tools such as *NanoPlot* (Coster 2025) are capable of visualizing read length distributions and base called quality scores, while *pycoQC* (Leger and Leonardi 2019) could generate interactive summaries and prepared QC metrics. Additionally, *Porechop_ABI* (bonsai., 2022) specifically focuses on detecting adapters along with barcode sequences, while the trimming and filtering instrument of *Nanop* (esteinig. GitHub - esteinig, nanoq 2023) could also be adopted in terms of ONT reads. More instruments like *MinIONQC* (roblanf., 2020) are famous for the fast and lightweight scripts enabling rapid and replicable comparisons from multiple flowcells’ data, and *ToulligQC* can be adaptable to both DNA and RNA sequences and is compatible with 1D^2^ runs as well (Genomique 2022; GenomiqueEns 2024).

Overall, the nanopore sequencing analytical pipeline has matured into a sophisticated framework supported by continual algorithmic and software innovation. These community-driven developments have steadily improved accuracy, scalability, stability, and accessibility across a wide range of applications.

## RNA modification detection from nanopore sequencing with various modeling frameworks

The emergence of advanced frameworks—especially statistical tools, ML, DL, and transformer-based LLMs—has fundamentally transformed the modification detection based on Nanopore Sequencing, allowing for real-time, accurate detection of modified bases and complicated analyses (Wang, et al. 2024, 2021; Petersen, et al. 2019). A collective overview of representative tools could be found in Table 1 and Supplementary Table 2.

**Table 1:** Summaries of common Nanopore (ONT) RNA Modification Detection Methods with utilized Model Types (Wang, et al. 2021)

Method	Modification Type (Partial)	Model Framework utilized (Partial)	Training Data	Quantification	Negative Control (UsageStage)	Feature
*CHEUI (CHEUI_solo, CHEUI_diff)*	m6A, m5C	two-stage Deep Learning method	In vitro	P	P (For CHEUI_diff)	Current
*DeepEdit*	Inosine	Neural Network (NN)	In vivo	P		Current
*DENA*	m6A	Bidirectional Long Short-Term Memory (BiLSTM)	In vivo	P		Current
*DiffErr*	m6A	Statistical Testing	N/A		P	BSE
*Dorado* (Base Calling-based Modification Detection)	m6A, Ψ (Updating)	Deep Learning (PyTorch)	NA			Current
*DRUMMER*	m6A	Statistical Testing	N/A		P	BSE
*ELIGOS*	Multiple	Fisher's Exact Tests, Statistical Testing	In vitro		P	BSE
*EpiNano (EpiNano-SVM, EpiNano-Error)*	m6A	Support Vector Machines (SVM), Regression	In vitro		P (For EpiNano-Error)	BSE
*IL-AD* (Base Calling-based Modification Detection)	m6A, m1A, m5C	incremental learning (IL), anomaly detection (AD)	In vitro	P		Current
*m1a-prediction*	m1A	Machine Learning	In vitro	P		Current
*m6ABasecaller* (Base Calling-based Modification Detection)	m6A	taiyaki, NanoRMS2(Machine Learning)	In vitro + In vivo	P		Current
*m6Anet*	m6A	Neural Network (NN), Multiple Instance Learning	In vivo	P		Current
*m6ATM*	m6A	Deep Neural Network (NN), WaveNet + Dual-stream multi-instance learning	In vitro	P		Current
*mAFiA, Ψ-co-mAFiA* (improved)	m6A, Ψ	RODAN-based neural network	In vitro	P		Current
*MINES*	m6A	Random Forest (RF)	In vivo			Tombo Output
*modCnet*	m5C, ac4C	Deep Learning Framework	In vitro	P		Current
*ModiDeC*	Multiple (m6A, Ψ, Gm, m1A, Inosine)	neural network, LSTM	In vitro	P		Current
*ModQuant*	Ψ	Machine Learning	In vitro	P		Current + BSE
*Nanocompore*	Multiple (m6A, Inosine, m5C, Ψ, m6,2A, m1G)	Bayesian Generative Models, Gaussian Mixture Model (GMM)	N/A		P	Current
*NanoDoc*	Multiple	Convolutional Neural Network (CNN), Deep One-Class Classification (DOC)	In vitro		P	Current
*NanoDoc2*	Multiple	Neural Network (NN), Clustering	In vitro		P	Current
*Nanom6A*	m6A	XGBoost (XGB)	In vitro	P		Current
*NanoMUD*	Ψ, m1Ψ	Bidirectional Long Short-Term Memory (BiLSTM)	In vivo	P		Current
*NanoNm*	Nm	XGBoost	In vitro	P		Current
*NanoPsu*	Ψ	Extremely Randomized Trees (EXT)	In vivo	P		BSE
*nanoRMS*	Ψ, Nm	Kmeans Clustering, K-nearest Neighbors (KNN)	In vivo	P		Current + BSE
*Nm-Nano*	Nm	XGBoost (XGB), Random Forest (RF)	In vivo			Current
*Penguin*	Ψ	Support Vector Machines (SVM), Neural Network (NN), Random Forest (RF)	In vivo			Current
*PsiNanopore*	Ψ	Comparative Analysis	In vitro	Semi-quantitative	P	BSE (U-to-C mismatch)
*pum6a*	m6A	Attention based framework, Positive and Unlabeled Multi-instance Learning	In vivo			Current
*RedNano*	m6A	deep residual networks (ResNet), CNN	In vivo			Current + BSE
*Remora*	Multiple	Neural Network (NN)	N/A	P		Current
*RNANO*	Multiple (m6A, m1A, m5C, m7G, ac4C, Nm, Ψ)	attention-enhanced multi-instance learning	In vivo	P		Current
*SingleMod*	m6A	Multiple Instance Regression (MIR)	In vivo	P		Current
*TandemMod*	Multiple (m6A, m5C, m7G, Ψ, Inosine)	Transferable Deep Learning framework	In vitro	P		Current + BSE
*Tombo*	Multiple	Statistical Analysis	N/A	P		Current
*xPore*	m6A	Bayesian Gaussian Mixture Model	N/A	P	P	Current
*Yanocomp*	m6A	Bayesian Generative Models, General Mixture Models (GMM)	N/A	P	P	Current

The table only summarizes partial information with utilized models or detailed frameworks; partial omission might occur during the process. In this table, the label “P” denotes presence. Additional information about Paper, GitHub and publication years could be found in Supplementary Table 2

Abbreviations: *Ψ* Pseudouridine, *m1Ψ* N1-methylpseudouridine, *Nm* 2´-O-methylation

Several tools, such as *Tombo* (nanoporetech 2020a, b; OxfordNanoporeTechnologies 2017a, b), *Nanocompore* (Leger 2019), *xPore* (GoekeLab 2021; Pratanwanich, et al. 2021), and *DiffErr* (bartongroup., 2020), have utilized clustering techniques or statistical testing rules for RNA modification detection procedures. The Bayesian Generative Models were the main modelling designs for *Nanocompore*, *xPore* and *Yanocomp* where the Gaussian Mixture Models (GMM) played an essential role (Zhao, et al. 2022). Moreover, tools of *Differr* (bartongroup., 2020) and *Drummer* (Abebe, et al. 2022) adopted G-tests based on the differential testing schemes, while *ELIGOS* (Jenjaroenpun, et al. 2020) conducted Fisher’s Exact tests for comparisons of error profiles across Direct RNA Sequencing (DRS) modified and control datasets (Furlan, et al. 2021; Zhao, et al. 2022).

Beyond statistical methods, some models have learnt ML or DL structures to identify RNA modifications like *Nanom6A* (Gaoyubang., 2025), *EpiNano* (Liu, et al. 2021; enovoa., 2025), *MINES* (YeoLab 2019), and *m6Anet* (GoekeLab 2023). Specifically, *Nanom6A* utilized an Extreme Gradient Boosting (XGBoost) model for m6A modification at single-base resolution according to nanopore DRS techniques (Gaoyubang., 2025; Gao, et al. 2021), while a Neural Network (NN) based structure called *m6Anet* was used to analyze transcriptome-wide quantification and identification of m6A with outperformed accuracy (GoekeLab 2023; Simpson, et al. 2017). In addition, the method of *MINES* leveraged Random Forest (RF) to detect m6A sites under the context of four sequences (AGACT, GGACA, GGACC, and GGACT) (Lorenz, et al. 2019; YeoLab 2019), and the instrument called *EpiNano* introduced Support Vector Machines (SVM) to distinguish modified bases such as m6A (Liu, et al. 2021; enovoa., 2025; Gao, et al. 2021; Simpson, et al. 2017; Liu, et al. 2019a, b). Moreover, tools like *NanoDoc* applied CNN and Deep One-Class Classification (DOC) (Ueda 2020), while improved *NanoDoc2* enabled detection of multiple RNA modifications by combining NN, and clustering algorithms (uedaLabR 2021). Furthermore, *mAFiA* (dieterich 2024a, b) and *Ψ-co-mAFiA* (dieterich 2024a, b) focused on m6A and Ψ using RODAN-based neural networks (Chan, et al. 2024), while *SingleMod* (Xieyy 2025; Xie, et al. 2025) applied Multiple Instance Regression (MIR) and *RedNano* (Derryxu., 2025; Ni, et al. 2024) utilized deep residual networks for m6A detections.

Numerous efforts have focused specifically on m6A, including *m6ATM* (Yu, et al. 2024) based on NN designs (Furlan, et al. 2021), *DENA* and *CHEUI* (comprna., 2021) emphasizing Nanopore DRS (Zhao, et al. 2022), *pum6a* (Liu, et al. 2025) adopting an Attention-based framework. Apart from that, great breakthroughs have also been made in detecting other modifications. *DeepEdit* (Chen, et al. 2023) was famous for its Inosine detection, while *TandemMod* (Yulab., 2024) made attempts at multiple detections like m6A, m5C, m7G, Ψ, and Inosine based on Transferrable DL frameworks. *Penguin* (Janga 2021) applied several ML models, including SVM, NN, and RF, showing its capability to identify Ψ sites with an accuracy of 93.38% in the Hek293 benchmark dataset (Janga 2021; Hassan, et al. 2022). Similarly, two supervised ML models of XGBoost and RF were found in *Nm-Nano* (Janga 2023) and demonstrated high accuracy (~ 88% for XGBoost and ~ 92% for RF) for 2′-O-methylation (Nm) detection (Janga 2023; Hassan, et al. 2024). Moreover, *NanoNM* (kaifuchenlab., 2024; Li, et al. 2024) detected Nm sites, *m1a-prediction* (Chen, et al. 2024; BernieeeX., 2023) focused on m1A modifications and *ModQuant* (wanunulab 2022; Amr, et al. 2024) identified Ψ sites using ML architectures. Meanwhile, tools like *nanoRMS* (novoalab 2022) focused on Nm and Ψ RNA modifications, especially the detection in cellular RNAs using unsupervised (i.e., K-means Clustering) and supervised classification (i.e., KNN: K-nearest Neighbors) (novoalab 2022; Hassan, et al. 2024; Begik, et al. 2021). Furthermore, *NanoMUD* (Zhang, et al. 2024a, b) utilized Bidirectional Long Short-Term Memory (BiLSTM) for Ψ along with N1-methylpseudouridine (m1Ψ), while tools of *NanoPsu* (Huang, et al. 2021; sihaohuanguc 2021) and *Psinanopore* (Tavakoli, et al. 2023; RouhanifardLab., 2025) also paid special attention on Ψ detection.

Recently, multi-modification detection has gained increasing attention with the development of robust deep learning frameworks such as *ModiDeC* (mem3nto 2025; Alagna, et al. 2025), which enabled the identification of multiple modifications such as m6A, Ψ, Gm, m1A, and Inosine. In parallel, the advanced *RNANO* (abhhba 2025; Wang, et al. 2025) model leveraged attention-enhanced multi-instance learning to detect a broader range of modifications, including m6A, m1A, m5C, m7G, ac4C, Nm, and Ψ.

One notable development is the emergence of base calling-based modification detection tools that could integrate modification prediction into the base calling process. For example, *m6ABasecaller* (novoalab 2023; Cruciani, et al. 2025) was introduced as a modification-aware base calling model capable of producing m6A predictions in individual reads during base calling, enabling high-resolution investigation into m6A deposition mechanisms. Similarly, the *IL-AD* (Wangziyuan 2023; Wang, et al. 2024) framework adapted base calling models for modification detection using incremental learning (IL) and Anomaly Detection (AD) strategies. Additionally, the official ONT tool *Dorado* (OxfordNanoporeTechnologies 2024a, b; nanoporetech 2022) has supported modified base calling for m6A and Ψ, with ongoing efforts to expand compatibility to other modifications such as m5C, Nm and Inosine.

Due to the large number of different methods, several conditions and criteria could be considered to find the most suitable method for specific detection tasks. For instance, some frameworks are trained on in vivo datasets (e.g., *MINES*, *m6Anet*, *NanoMUD*), whereas others including *Nanom6A*, *Psinanopore* and *TandomMod* utilize in vitro data. Such careful selection might depend on the analytical emphases, either on native biological contexts reflected better in vivo environments, or the engineered sequence designs under controlled vitro trials. Moreover, practical applicability often depends on whether negative controls are available, meaning methods like *Nanocompore*, *DiffErr*, *DRUMMER*, *Psinanopore* are intrinsically comparative and unsuitable without matched controls. Similarly, the output formats should be tailored to the study objectives, with models like *m6Anet*, *Nanom6A*, *NanoMUD* yielding quantitative modification-rate estimates, and the contradictory side only providing binary measures. Furthermore, feature designs might vary across frameworks, with many of them using signal-level summaries (i.e., mean, standard deviation and dwell time) while others like *PsiNanopore* relying on U-to-C mismatch which belonging to the Base calling Error (BSE) features. Finally, computational requirements must be considered, where DL and especially LLM-augmented pipelines typically require substantial GPU resources, whereas lighter statistical methods seem more accessible in some resource-constrained settings.

The practical utility of these tools is reflected in several biological applications. For example, Price et al. employed tools of *DRUMMER* and *Nanocompore* to identify m6A sites and further demonstrated that m6A methylation could enhance the splicing efficiency within adenovirus late transcriptional units (Price, et al. 2020). Moreover, the *m6Anet* framework was utilized with additional detection of allele-specific m6A modifications (Park and Cenik 2024). Furthermore, *NanoMUD* was applied to predict RNA Ψ and m1 Ψ modifications and could facilitate the design and quality control of chemically modified mRNA vaccines (Zhang, et al. 2024a, b). Together, such examples have underscored how diverse computational approaches could be effectively leveraged to explore distinct biological questions and investigate mechanistic insights within the field of RNA modification.

Collectively, these frameworks or designs, from initial statistical or HMM-based trials to further ML or DL added models, and to recent pre-trained LLM applications, have gradually undergone a technical or biomedical revolution under complex biological scenarios (Ji, et al. 2021; Wang, et al. 2021). These innovations have significantly improved the sensitivity, scalability, and interpretability of base modification analysis, which could open the door to a more comprehensive, accurate, and mature world of nanopore sequencing (Wang, et al. 2021).

## Benchmark analysis of RNA modification detection of m6A in nanopore sequencing

To systematically evaluate the performance of current computational tools for detecting RNA modifications from nanopore sequencing data, we conducted a benchmark analysis specifically targeting m6A. Five representative detection tools—*m6Anet, MINES, nanom6A, DRUMMER*, and *DiffErr*—were independently applied to two publicly available human transcriptomic datasets: HEK293_WT and HMEC_WT (WT: Wild Type). These RNA samples were from GEO under accession number GSE132971. Only common chromosomes including Chr1-22, ChrX, ChrY and ChrM were included, and probability of 0.5 was utilized for cutoff purposes.

For standardized comparison, we employed the NGS-benchmarked database as the reference standard (Zhang, et al. 2022a, b) (Supplementary Table 3), which aggregates transcriptome-wide m6A sites derived from 9 different NGS platforms with single-base resolution. Based on the number of times documented by different technologies, m6A sites were categorized into four confidence levels: high-confidence (1,243 sites; supported ≥ 4 times), medium-confidence (6,792 sites; supported 3 times), low-confidence (26,297 sites; supported 2 times), and very low-confidence (99,357 sites; supported once).

**Fig. 4:**
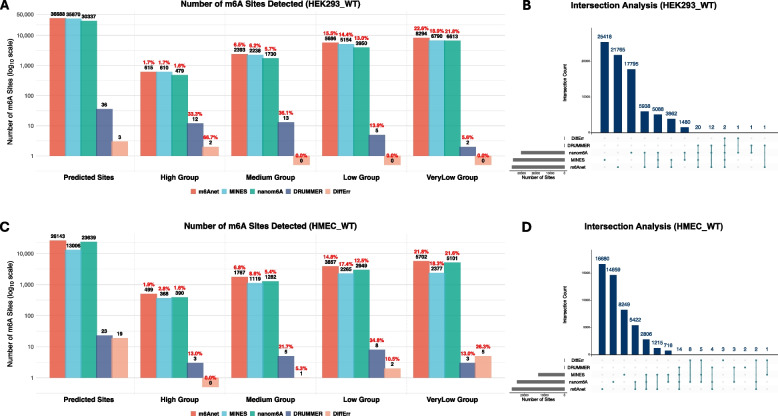
m6A Detection using 5 different tools of *m6Anet*, *MINES*, *nanom6A*, *DRUMMER* and *DiffErr*. Total Predicted m6A sites detected, and number of m6A sites in 4 categories of different confidence levels (High/Medium/Low/Very Low groups) in HEK293_WT (**A**) and HMEC_WT (**C**). Upset plots representing the number of overlapped m6A sites detected by 5 different tools in HEK293_WT (**B**) and HMEC_WT (**D**)

In the HEK293_WT, we evaluated the number and distribution of predicted m6A sites identified by five representative detection tools. As shown in Fig. 4A (“Predicted Sites” column), *m6Anet* identified the highest number of m6A sites (*n* = 36,688), followed by *MINES* (*n* = 35,870) and *nanom6A* (*n* = 30,337). In contrast, *DRUMMER* and *DiffErr* reported only a limited number of sites, indicating a more conservative detection profile.

To further investigate the consistency and overlap among these tools in HEK293_WT, we performed an intersection analysis across the five models (Fig. 4B). Among them, 5,088 sites were commonly detected by the three leading tools (*m6Anet, MINES*, and *nanom6A*), suggesting a robust subset of high-confidence predictions. Additionally, 5,938 sites were co-detected by *m6Anet* and *nanom6A*, while 3,862 sites overlapped between *m6Anet* and *MINES*., and 1,480 sites co-existed in *nanom6A* and *MINES*.

To evaluate detection performance in the context of reference confidence, we mapped the predicted sites in HEK293_WT to the NGS-benchmarked database and stratified them into four groups based on confidence levels. For the three major tools—*m6Anet, nanom6A*, and *MINES*—a substantial proportion of predictions aligned to the Low and Very Low categories (Fig. 4A). For instance, *m6Anet* detected 5,686 sites (15.5%) in Low group and 8,294 sites (22.6%) in Very Low group. Similarly, *nanom6A* identified 3,950 (13.0%) and 6,613 (21.8%) sites in Low and Very Low categories, with an additional 1,730 (5.7%) in the Medium and 479 (1.6%) in the High group. And *MINES* yielded 6,790 (18.9%) Very Low, 5,154 (14.4%) Low, 2,238 (6.2%) Medium, and 610 (1.7%) High-confidence matches correspondingly. In contrast, *DRUMMER* detected only 36 sites with a higher proportion mapping to more confident categories, specifically 13 (36.1%) to Medium and 12 (33.3%) to High. *DiffErr* reported only 3 sites in total, among which 2 sites (66.7%) belonged to the High-confidence group. Additionally, it is admitted that a small subset of detected sites from all tools could not be assigned to any confidence category due to missing annotations in the reference dataset as well. Detailed model confusion metrics as well as indicator of precision, recall and F1 were collected in Supplementary Table 4.

A similar analysis was conducted on the HMEC_WT dataset to assess tool performance across a distinct human transcriptomic background. As shown in Fig. 4C, m6Anet*, MINES*, and *nanom6A* again outperformed the other tools in terms of total number of predicted m6A sites, reporting 26,143, 13,006, and 23,639 sites accordingly. These trends were largely consistent with those observed in HEK293_WT, confirming the broad applicability and sensitivity of these three models. *DRUMMER* and *DiffErr* still remained highly conservative, identifying only 23 and 19 sites in HMEC_WT dataset.

Intersection analysis (Fig. 4D) in HMEC_WT revealed 2,806 m6A sites commonly predicted by *m6Anet, MINES*, and *nanom6A* in HMEC_WT, slightly smaller than the 5088 sites shared in HEK293_WT. However, 14 sites in HMEC_WT were observed to co-exist in four major tools except for *DiffErr*, and *DiffErr* seemed to detect more m6A sites (*n* = 19) in HMEC_WT than previous HEK293_WT dataset (*n* = 3). Additionally, the overlaps between *m6Anet* and *nanom6A* (5,422 sites), between *m6Anet* and *MINES* (1,215 sites), and between *nanom6A* and *MINES* (716 sites) further supported the internal consistency among these tools.

When predictions in HMEC_WT were mapped to the NGS-benchmarked reference, the confidence group distribution remained skewed toward lower-confidence categories for most tools. For instance, *m6Anet, MINES* and *nanom6A* identified 5,702 (21.8%), 2377 (18.3%) and 5101 (21.6%) m6A sites in Very Low group, along with 3857 (14.8%), 2265 (17.4%) and 2949 (12.5%) in the Low category. *DiffErr*, although limited in total predictions, still had the majority distribution in lower confident group (5 sites, 26.3% for Very Low; 2 sites, 10.5% for Low). Moreover, *DRUMMER* maintained a higher percentage of matches within the Low and Medium group (8 sites, 34.8% for Low; 5 sites, 21.7% for Medium) as well (Fig. 4C). Similarly, several NA values were also unavoidable when applying such confidence classification. Additional confusion variables and other evaluating elements were displayed in Supplementary Table 5.

Furthermore, Supplementary Fig. 1A presented the proportions of overlapping and non-overlapping predicted m6A sites. The Low and Very Low groups exhibited higher overlap percentages, with *m6Anet* achieving relatively greatest number of true positives among the three prevalent models (*m6Anet*, *MINES*, and *nanom6A*). Consistently, the Very Low confidence group, accounting for 74.3% of total sites, dominated the NGS reference (Supplementary Fig. 1B), and detailed precision and recall curves were shown in Supplementary Fig. 1C as well.

Collectively, the findings of m6A benchmark revealed markedly divergent detection landscapes across different tools. Such discrepancies seemed to be prevalent in other modifications or scenarios as well, which would underscore the need for further methodological advancements such as detection accuracy, model robustness and cross-tool consistency.

## Benchmark analysis of RNA modification detection of pseudouridine in nanopore sequencing

To systematically evaluate the performance of pseudouridine (Ψ) detection from nanopore sequencing data, we also focused on three representative algorithms—*Penguin*, *Nanomud*, and *Nanopsu*. These tools were independently applied to the HEK_WT dataset for the benchmark analysis, and only common chromosomes (Chr1–22, ChrX, ChrY, and ChrM) were considered in the analysis. Similar as m6A, Ψ also had a NGS dataset for reference (https://rna.sysu.edu.cn/rmbase3) where different site information could be extracted accordingly.

**Fig. 5:**
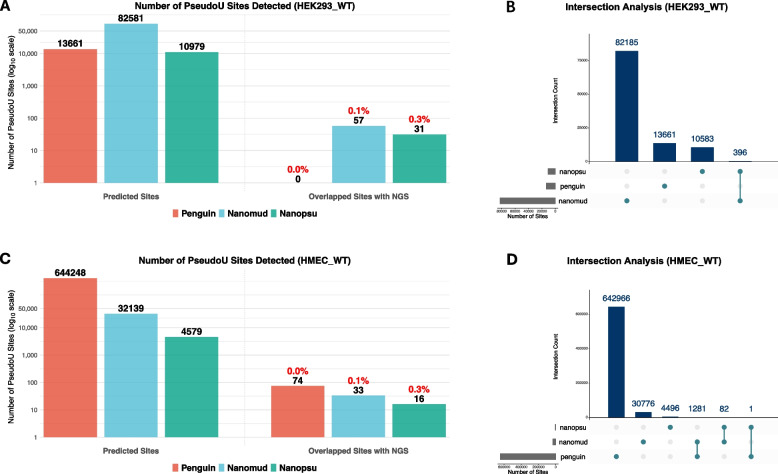
Pseudouridine (Ψ) Detection using 3 different tools of *Penguin*, *NanoMUD* and *Nanopsu*. Total Predicted Ψ sites detected, and number of Ψ sites in 4 categories of different confidence levels (High/Medium/Low/Very Low groups) in HEK293_WT (**A**) and HMEC_WT (**C**). Upset plots representing the number of overlapped Ψ sites detected by 5 different tools in HEK293_WT (**B**) and HMEC_WT (**D**)

According to Fig. 5A, the total number of predicted Ψ sites varied substantially across tools, with *Nanomud* identifying the largest number (*n* = 82,581), followed by *Nanopsu* (*n* = 10,979) and *Penguin* (*n* = 13,661). To further assess prediction reliability compared to NGS-based reference, the proportion of overlapped sites with NGS was extremely limited for all tools with 57 sites (0.1%) for *Nanomud* and 31 (0.3%) for *Nanopsu*. Moreover, Fig. 5B showed the intersection analysis where the overlap across the models was minimal, and only 396 sites commonly shared by both *Nanomud* and *Nanopsu*.

In terms of dataset of HMEC_WT, *Penguin* identified the largest number of Ψ sites (*n* = 644,248), followed by *Nanomud* (*n* = 32,139) and *Nanopsu* (*n* = 4,579). When mapped to the NGS-based reference, the proportion of overlapped sites remained extremely limited for all tools: only 74 sites for *Penguin*, 33 sites (0.1%) for *Nanomud*, and 16 sites (0.3%) for *Nanopsu* (Fig. 5C). In terms of intersection analysis for Ψ, *Penguin* and *Nanomud* had 1281 sites in common, whereas *Nanopsu* shared 82 sites with *Nanomud* and had only 1 overlapped site with *Penguin* (Fig. 5D). Detailed metrics for Ψ benchmark were in Supplementary Table 6.

These findings reveal considerable discrepancies between NGS and Nanopore detection tools, with inconsistencies in Ψ identification being even more pronounced than m6A modifications. Therefore, this substantial lack of consensus has highlighted the divergent NGS and Nanopore detection algorithms towards Ψ, further underscoring the need for improved cross-tool consistency and methodological refinement in RNA modification analysis.

## Biological significance and comparative evaluation of RNA modification detection

### Functional meaning of different types of modifications

Chemical modifications of nucleic acids, both for RNA and DNA, have played pivotal roles in regulating gene expression and maintaining cellular homeostasis. Among these, m6A is recognized as the most prevalent internal modification in eukaryotic messenger RNA (mRNA). It regulates key processes such as splicing, nuclear export, translation, and mRNA stability, thereby influencing stem cell differentiation, circadian rhythms, and tumorigenesis (Jiang, et al. 2021). Dysregulation of m6A and its associated proteins has been implicated in a range of diseases, including nonalcoholic fatty liver disease, azoospermia (Chen, et al. 2020), heart failure (Berulava, et al. 2019), and multiple cancers (e.g., acute myelocytic leukemia (AML), brain tumor, reproductive system malignancies (Liu, et al. 2020)), highlighting its potential as a therapeutic target (Jiang, et al. 2021). Apart from human systems, studies in species like Arabidopsis, rice, cotton, and tomato have demonstrated the involvement of m6A as “writers” and “erasers” in regulating gene expression (Cai, et al. 2024), emphasizing the essential role of m6A in plant adaptation to abiotic stresses. Importantly, such insights could open new avenues for crop improvement, as m6A-targeted genome and transcriptome engineering would enable the development of stress-tolerant plant varieties through precise modification detection and manipulation (Cai, et al. 2024).

Beyond m6A, other RNA modifications could also exert essential effects. For example, m5C has been proven to serve as the nuclear export regulator and protein translation modulator, with its distribution within the mRNA imparting distinct effects on mRNA functions (Trixl and Lusser 2018; Schümann, et al. 2020). Moreover, m7G together with internal m6Am, have been implicated in the regulation of global RNA alternative splicing in human diseases (Qiu, et al. 2023; McGinty, et al. 2008). In addition, Ψ modification is demonstrated to enhance RNA stability, modulate transcriptional dynamics and improve base-stacking as well. Similarly, m1A has also been reported to affect mRNA structural stability by disrupting Watson–Crick base pairing and to increase accessibility of translation machinery through alternations in secondary structure of the mRNA 5’UTR regions (Qiu, et al. 2023).

### Comparative evaluation of modification detection strategies

Detection of nucleotide modifications has evolved through both sequencing-based and non-sequencing strategies. Non-sequencing techniques — including two-dimensional thin-layer chromatography (2D-TLC), dot blot, and liquid chromatography-mass spectrometry (LC–MS) — enable accurate quantification of modifications but lack sequence-specific information (Zhang, et al. 2022a, b). Specifically, 2D-TLC distinguishes nucleotides based on their differential mobilities in the solvent and requires only a small amount of RNA (typically 50–200 ng) owing to its high sensitivity (Zhang, et al. 2022a, b; Pobłocka-Olech, et al. 2025). The dot blot assay, which relies on modification-specific antibodies, has been widely applied to diverse RNA species such as non-coding RNAs and mRNAs (Zhang, et al. 2022a, b), although this method lacks absolute quantification and locus information (Zhang, et al. 2022a, b). In contrast, LC–MS has become a benchmark technique for both detection and quantification of RNA modifications in spite of the requirement of sophisticated instrumentation such as high-performance liquid chromatography (HPLC) coupled with mass spectrometry (Zhang, et al. 2022a, b; Manasses, et al. 2018).

The NGS methods, including the well-known *Illumina* platform, rely on a variety of strategies to infer RNA modification sites. These include protocols of Antibody-based (e.g., MeRIP, miCLIP-seq, hMeRIP, acRIP-seq, m6Am-seq), Chemical-assisted methods (e.g., BoRed-seq, ICE-seq, m6A-SEAL-Seq, RBS-seq), Enzyme/Protein-assisted techniques (e.g., AZA-IP, DART-seq, MAZTER-seq), and Direct Sequencing approaches (e.g., MeTH-seq, m1A-quant-seq) to indirectly enable transcriptome-wide profiling (Zhang, et al. 2022a, b; Xuan, et al. 2013).

More recently, advanced NGS-based approaches have achieved remarkable improvements in both accuracy and quantitative capability for RNA modification mapping, and two representative examples are *GLORI* (glyoxal and nitrite-mediated deamination of unmethylated adenosines) and *eTAM-seq* (evolved TadA-assisted N6-methyladenosine sequencing). In *GLORI* (Sun, et al. 2025), unmodified adenosines are selectively deaminated to Inosines (read as guanorines during reverse transcription), whereas m6A remain unreactive and are read as adenosines, which further enables unbiased and absolute quantification of m6A at single-base resolution (Sun, et al. 2025; Liu, et al. 2023). *GLORI* (Liucongcas., 2022) has exhibited substantially superior analytical accuracy and reproductivity, despite its relatively expensive cost compared with enrichment methods such as MeRIP (Liu, et al. 2023). Similarly, *eTAM-seq* (shunliubio 2022) employs global adenine deamination through the TadA-assisted mechanism, allowing detection of m6A as persistent adenosines while unmethylated adenosines are converted to Inosine accordingly (Xiao, et al. 2023). Such enzyme-assisted strategy provides highly specific detection and quantitative assessment of m6A, even from very limited RNA input samples, thereby further extending its applicability (Xiao, et al. 2023).

Although different types of NGS-based detection technologies are widely applied for transcriptome-wide profiling, their short-read constraints (typically 50–300 bp) and indirect detection mechanisms might restrict the comprehensive characterization of RNA modification landscapes (Zhang, et al. 2022a, b). In contrast, the third-generation SMRT sequencing (PacBio) could offer identification of modifications by monitoring polymerase kinetics without further sample preparations (Rhoads and Au 2015). This approach allows direct detection of modifications like 5mC and 6 mA, and can resolve methylation patterns even in highly repetitive genomic regions (Flusberg, et al. 2010). Similarly, ONT platform employs direct long-read sequencing by measuring ionic current fluctuations as molecules pass through a biological nanopore, which has offered several distinct advantages over other modification detection methods.

To be specific, unlike non-sequencing tools which are often modification-specific and indirect, nanopore sequencing enables direct and real-time detection of modifications with single-nucleotide resolution (Zhang, et al. 2022a, b; Garalde, et al. 2016; Burgess 2017). Then compared with NGS techniques such as Illumina, which requires additional reverse transcription and PCR amplification, nanopore sequencing could sequence in a direct manner and decrease the biases resulting from such steps (Zhang, et al. 2022a, b). In addition, short-read sequencing including NGS require RNA fragmentation, leading to multimapping issues and isoform ambiguity, whereas nanopore long-read sequencing could capture full-length transcripts and enable the precise isoform identification and quantification (OxfordNanoporeTechnologies 2024a, b). Furthermore, in comparison with PacBio SMRT sequencing, the ONT method supports a broader range of modifications and enables single-molecule native RNA analysis, making it particularly well-suited for comprehensive epigenetic and epitranscriptomic studies (Flusberg, et al. 2010; Stephenson, et al. 2022; Garalde, et al. 2016).

### Assessment on base calling-based modification detection approaches

Several base calling-based modification detection strategies such as Dorado, m6ABasecaller, IL-AD have also been carried out, and different advantages have also emerged. For instance, these models could fully independent per-read predictions without additional procedures for statistical tests or calculations (Cruciani, et al. 2025; Wang, et al. 2024; Begik, et al. 2021). Moreover, RNA modifications are detected de novo during the base calling step, possibly bypassing computationally intensive processes such as resquiggling, feature extraction, and post hoc statistical analysis (Cruciani, et al. 2025; Wang, et al. 2024; Begik, et al. 2021; Acera Mateos, et al. 2022). Importantly, under most circumstances, these strategies can operate without the need for control samples and are not constrained by specific k-mers or prior knowledge of motifs (Cruciani, et al. 2025; Wang, et al. 2024), allowing for broader applicability across diverse datasets.

Despite their promising potential, the development of these modification-based base calling strategies might face some considerable challenges, mainly due to the lack of sufficient high-quality training datasets with confidently labeled modification status (Cruciani, et al. 2025). Specifically, such effective models require training data that include precise ground truth annotations indicating the exact presence or absence of modifications at specific nucleotide positions (Cruciani, et al. 2025). Beyond positional information, it is also important to have read information corresponding to modifications to ensure accurate per-read labeling (Cruciani, et al. 2025). These relatively strict requirements might pose substantial obstacles, making the development and construction of such models particularly difficult.

## Challenges and future directions of nanopore-based modification detection

Despite the transformative potential of nanopore sequencing, several technical and analytical challenges might hinder its broader application, yet these challenges also define the directions for future innovations.

A primary limitation lies in the accurate identification of complex, overlapping and multiple modifications (Warburton and Sebra 2023; Petersen, et al. 2019), which often produce subtle, context-dependent perturbations in the ionic signal that are difficult to resolve using conventional models (Petersen, et al. 2019; Amarasinghe, et al. 2020). Although advanced frameworks such as *TandemMod* (Yulab., 2024) have incorporated transfer learning and de novo training to support multi-modification detection, signal variability across different base types and complicated sequence contexts would still remain a significant hurdle (Yulab., 2024; Wu, et al. 2024).

Another persistent challenge is the relatively high sequencing error rate of nanopore technology. In spite of various attempts in accuracy enhancement including adaptive base calling or refined pore chemistry, the benchmarked error rate of Illumina short-read sequencing (0.1–1%) seems difficult to achieve by our ONT approaches (Zhang, et al. 2022a, b; Wang, et al. 2021). Since some computational pipelines infer modifications from base calling discrepancies or signal deviations, such intrinsic error levels might hinder reliable modification identifications accordingly (Zhang, et al. 2022a, b).

In addition, a practical limitation of ONT-based DRS lies in the relatively large RNA input requirement, which remains necessary to obtain robust throughput and signal quality (Zhang, et al. 2022a, b). According to the official ONT documentation, the SQK-RNA002 (OxfordNanoporeTechnologies 2018) workflow recommends 50 ng of poly(A)-tailed RNA or 500 ng of total RNA in 9 µL, whereas the updated SQK-RNA004 (OxfordNanoporeTechnologies 2023a, b) protocol specifies 300 ng of poly(A) RNA or 1 µg of total RNA in 8 µL for material preparation accordingly. These input requirements might substantially pose a major barrier for low-input samples such as clinical biopsies, single-cell samples (Picelli 2016; Huang, et al. 2018) or some early developmental specimens (Olds, et al. 1973), where their RNA yields are sometimes in the nanogram or even sub-nanogram range.

Beyond input limitations, cost-effectiveness and computational demand remain major concerns. For instance, the throughput of nanopore RNA sequencing is relatively low (1–3 Gb per flow cell), while the high cost of direct RNA sequencing, together with the requirement for high coverage (≥ 30X) to ensure accurate modification detection (Leger, et al. 2021; Pratanwanich, et al. 2021), would further increase the burden (Zhang, et al. 2022a, b). Meanwhile, the adoption of advanced computational models—often requiring high-end GPUs (Loose, et al. 2016), large memory, and extended training time—further increases infrastructure demands, posing challenges for some real-time and clinical implementations (Wang, et al. 2021; Liu, et al. 2019a, b; Pagès-Gallego and de Ridder 2023).

Nevertheless, these ongoing challenges have stimulated rapid methodological evolution, and future developments are expected to focus on reducing input requirements, improving pore chemistry, and enhancing signal processing to achieve higher accuracy and sensitivity, particularly for scarce or clinically derived RNA samples. The adoption of multiplexing may partially alleviate input constraints by pooling multiple RNA preparations, although this approach might reduce sequencing yield per individual sample. Recently, methodological advances have suggested the technical feasibility of DRS multiplexing, which would potentially represent a practical strategy to balance throughput and input to some extent (van der Toorn, et al. 2025; Smith, et al. 2020). In the meantime, the implementation of low-input and amplification-free protocols, coupled with improved adapter design or optimized motor proteins, may substantially expand the accessibility of nanopore-based epitranscriptomic studies as well (OxfordNanoporeTechnologies 2023a, b).

Equally promising is the improvement toward real-time analysis with modification-aware frameworks aimed at reshaping the nanopore sequencing landscape. Tools such as *SquiggleNet* have enabled real-time molecule classification and selective sequencing, allowing users to eject unwanted molecules (Bao, et al. 2021). Similarly, *Sigmap* has made breakthroughs by converting reference genomes to signals and enabling real-time mapping (haowenz., 2020). These advances, usually accompanied by GPU-accelerated computation and improved detection technologies, are already in development, and continued progress in this field would promise broader adoption of sequencing and time-efficient frameworks (Wang, et al. 2021; Bao, et al. 2021).

In parallel, ongoing algorithmic advances are expanding the modification spectrum detectable by nanopore platforms as well. Whereas earlier efforts primarily targeted the prevalent m6A and 5mC, more recent methods, including *Remora*, *Penguin* and *TandemMod,* have begun to explore a broader range of bases such as Ψ, Inosine and m7G (OxfordNanoporeTechnologies 2022; Hassan, et al. 2022; Yulab., 2024). Such ability to jointly detect multiple modifications within a single sequencing run would represent a promising direction for comprehensive epigenetic and epitranscriptomic profiling (Wang, et al. 2021). This progress will probably be facilitated by advances in model development, particularly through the integration of LLM and ML/DL-based architectures capable of capturing complex signal patterns and sequence contexts (Wang, et al. 2021; Zhang, et al. 2022a, b). For instance, the incorporation of Ensemble Learning by organically combining several frameworks, Transfer Learning towards rare or newly-emerged samples, and Unsupervised/Semi-Supervised Learning about anomaly detection (Wang, et al. 2021; Shendure, et al. 2017; Wu, et al. 2024), would potentially become emerging frontiers and focal points of innovation in the foreseeable future.

## Summary

This review has traced the evolution of sequencing technologies, from the first-generation Sanger method and NGS short-read platforms to TGS long-read systems, with a particular emphasis on the ONT strategy, which enables direct, real-time, and single-molecule sequencing. Then, the core analytical pipeline of nanopore sequencing has been dissected in detail, highlighting representative tools and methodologies across each stage of the workflow.

Furthermore, we surveyed a range of computational frameworks developed for RNA modification detection using nanopore sequencing, ranging from statistical approaches, ML and DL models, to emerging architectures incorporating transformers and attention mechanisms. Further benchmark analyses including 5 tools for m6A and 3 tools for Ψ were conducted on HEK293_WT and HMEC_WT respectively, and great disparity was presented across different detection tools.

Then the functional significance of specific nucleotide modifications has been illustrated, alongside comparative evaluations of existing detection strategies, with ONT-specific advantages clearly emphasized. In addition to the illustration of current technical challenges, we have also discussed the prospective research directions, which could collectively hold promise for broader and more accurate modification profiling and enhance the performance and applicability of nanopore sequencing.

## Supplementary Information

Supplementary Material 1.

Supplementary Material 2.

## Data Availability

The datasets used in our studies include two publicly available human transcriptomic datasets of HEK293_WT and HMEC_WT (WT: Wild Type), which are available in the Gene Expression Omnibus (GEO) under the accession number GSE132971. Additional benchmarking datasets utilized in our studies are obtained from these two links: 10.1016/j.ymeth.2022.04.003 and https://rna.sysu.edu.cn/rmbase3.
