# Supplementary figures and images for: Nanopore direct RNA sequencing for RNA modification analysis: workflow assessment and computational tool benchmarking
Source: Adv Biotechnol (Singap). 2026 Mar 10;4(1):9. doi: 10.1007/s44307-025-00093-5 (PMC12976157; doi:10.1007/s44307-025-00093-5)

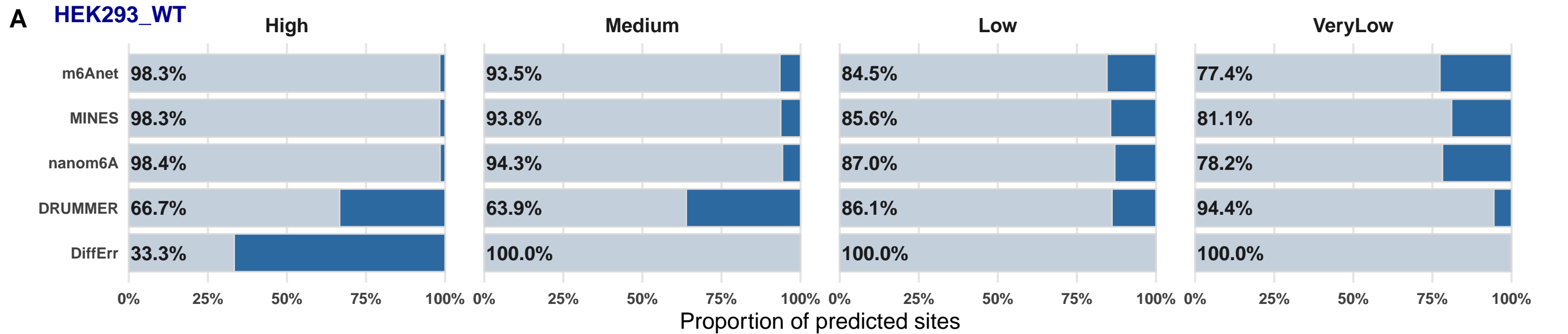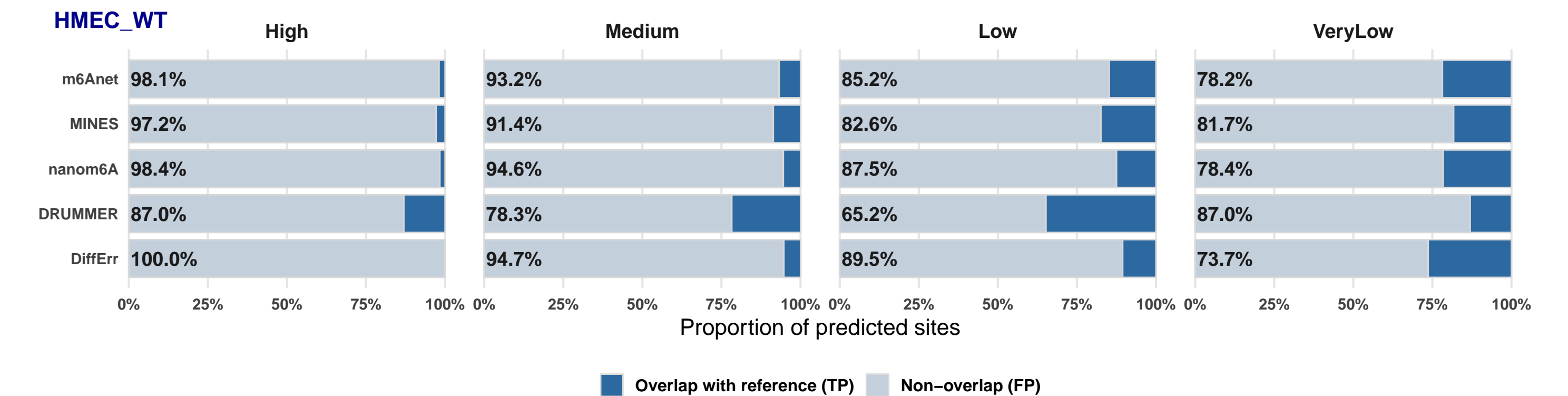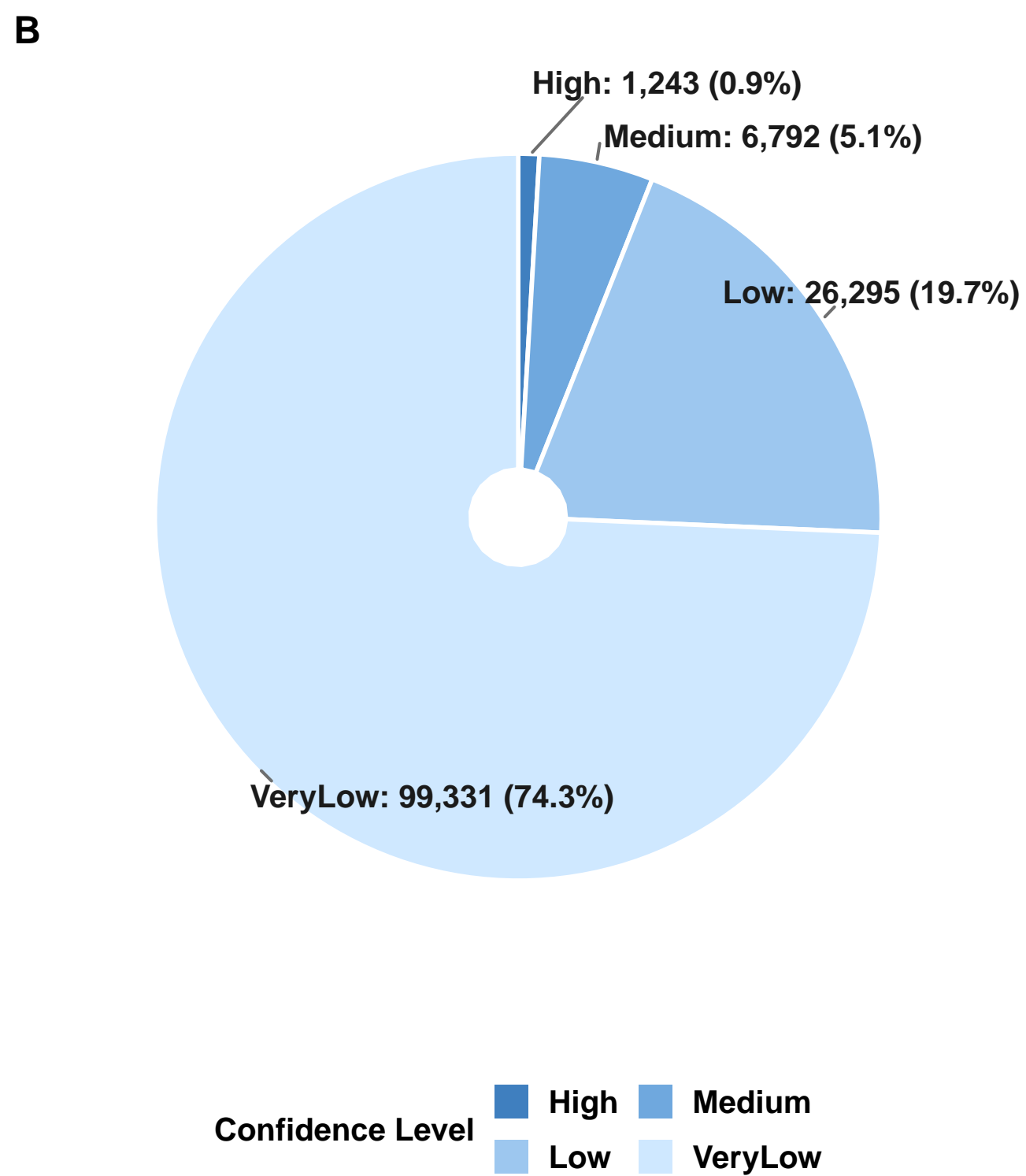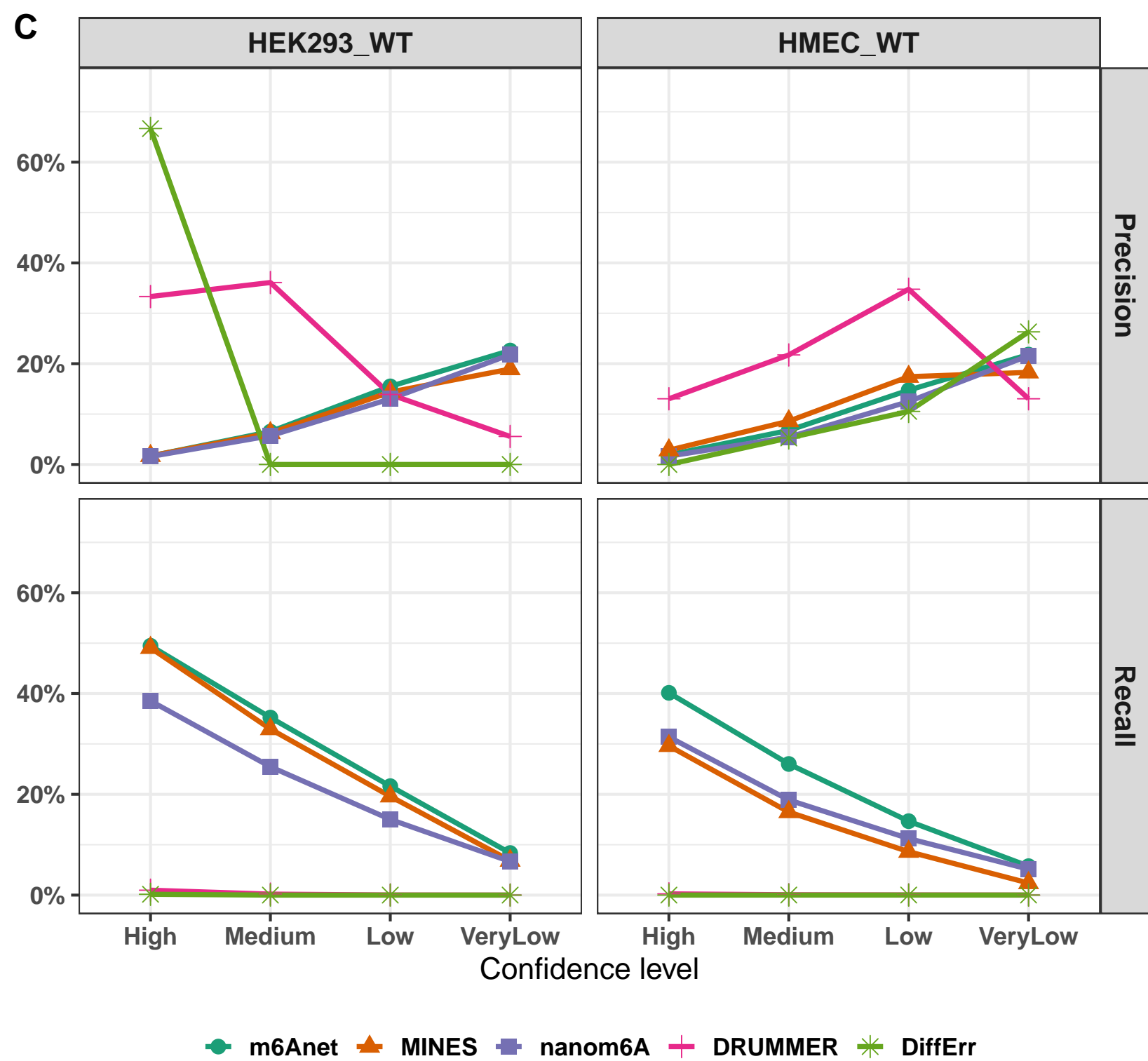

Supplement: Supplementary file 1 — Supplementary Material 1. [file 44307_2025_93_MOESM1_ESM.pdf]
