# Supplementary material for: Nanopore direct RNA sequencing for RNA modification analysis: workflow assessment and computational tool benchmarking
Source: Adv Biotechnol (Singap). 2026 Mar 10;4(1):9. doi: 10.1007/s44307-025-00093-5 (PMC12976157; doi:10.1007/s44307-025-00093-5)
Supplement: Supplementary file 2 — Supplementary Material 2. [file 44307_2025_93_MOESM2_ESM.docx]

**Nanopore Direct RNA Sequencing for RNA Modification Analysis: Workflow Assessment and Computational Tool Benchmarking**

**Supplementary Materials**

**Supplementary Table 1** Detailed model architecture of several Third-Party Base Calling Tools^42^

| **Tools** | **Architecture** | | |
| --- | --- | --- | --- |
|  | **Convolution** | **Encoder** | **Decoder** |
| **MinCall** | ResNet CNN | / | CTC |
| **CausalCall** | Causal Dilated CNN | / | CTC |
| **URNano** | Unet | GRU | CE |
| **SACall** | CNN | Transformer | CTC |
| **CATCaller** | CNN | Lite Transformer | CTC |
| **Bonito** | CNN | LSTM | CTC - CRF |
| **Halcyon** | Inception CNN | LSTM | ATT/LSTM - CE |

**Note for Abbreviations:** ATT (Attention), CE (Cross Entropy), CNN (Convolutional Neural Network), CRF (Conditional Random Field), CTC (Connectionist Temporal Classification), GRU (Gated Recurrent Unit), LSTM (Long Short-Term Memory

**Supplementary Table 2** Summaries of common **Nanopore (ONT) RNA Modification Detection** Methods with utilized Model Types^32^ (Remaining)

| **Method** | **Modification Type (Partial)** | **Year of Publication** | **Paper Source** | **Github Link** |
| --- | --- | --- | --- | --- |
| ***CHEUI (CHEUI_solo, CHEUI_diff)*** | m6A, m5C | 2024 | Prediction of m6A and m5C at single-molecule resolution reveals a transcriptome-wide co-occurrence of RNA modifications | <https://github.com/comprna/CHEUI> |
| ***DeepEdit*** | Inosine | 2023 | DeepEdit: single-molecule detection and phasing of A-to-I RNA editing events using nanopore direct RNA sequencing | <https://github.com/weir12/DeepEdit> |
| ***DENA*** | m6A | 2022 | DENA: training an authentic neural network model using Nanopore sequencing data of Arabidopsis transcripts for detection and quantification of N6-methyladenosine on RNA | <https://github.com/weir12/DENA> |
| ***DiffErr*** | m6A | 2020 | A tool for detecting modifications from Nanopore DRS errors using a low modification control | https://github.com/bartongroup/differr_nanopore_DRS |
| ***Dorado* (Base Calling-based Modification Detection)** | m6A, Ψ (Updating) | Updating | ONT Dorado | <https://github.com/nanoporetech/dorado?tab=readme-ov-file#modified-basecalling> |
| ***DRUMMER*** | m6A | 2022 | DRUMMER-rapid detection of RNA modifications through comparative nanopore sequencing | <https://github.com/DepledgeLab/DRUMMER> |
| ***ELIGOS*** | Multiple | 2020 | Decoding the epitranscriptional landscape from native RNA sequences | <https://gitlab.com/piroonj/eligos2> |
| ***EpiNano (EpiNano-SVM, EpiNano-Error)*** | m6A | 2019 | Accurate detection of m6A RNA modifications in native RNA sequences | <https://github.com/novoalab/EpiNano> |
| ***IL-AD* (Base Calling-based Modification Detection)** | m6A, m1A, m5C | 2024 | Adapting nanopore sequencing basecalling models for modification detection via incremental learning and anomaly detection | <https://github.com/wangziyuan66/IL-AD> |
| ***m1a-prediction*** | m1A | 2024 | Quantitative profiling N1-methyladenosine (m1A) RNA methylation from Oxford nanopore direct RNA sequencing data | https://github.com/BernieeeX/m1a-prediction |
| ***m6ABasecaller* (Base Calling-based Modification Detection)** | m6A | 2025 | De novo basecalling of RNA modifications at single molecule and nucleotide resolution | [https://github.com/novoalab/m6ABasecaller  https://github.com/novoalab/nanoRMS2](https://github.com/novoalab/m6ABasecaller) |
| ***m6Anet*** | m6A | 2021 | Detection of m6A from direct RNA sequencing using a Multiple Instance Learning framework | <https://github.com/GoekeLab/m6anet> |
| ***m6ATM*** | m6A | 2024 | m6ATM: a deep learning framework for demystifying the m6A epitranscriptome with Nanopore long-read RNA-seq data | <https://github.com/poigit/m6ATM> |
| ***mAFiA, Ψ-co-mAFiA* (improved）** | m6A, Ψ | 2024 | Detecting m6A at single-molecular resolution via direct RNA sequencing and realistic training data | [https://github.com/dieterich-lab/mAFiA  https://github.com/dieterich-lab/psi-co-mAFiA](https://github.com/dieterich-lab/mAFiA) |
| ***MINES*** | m6A | 2020 | Direct RNA sequencing enables m6A detection in endogenous transcript isoforms at base-specific resolution | <https://github.com/YeoLab/MINES.git> |
| ***modCnet*** | m5C, ac4C | 2025 | Simultaneous profiling of ac4C and m5C modifications from nanopore direct RNA sequencing | <https://github.com/yulab2021/modCnet> |
| ***ModiDeC*** | Multiple(m6A, Ψ, Gm, m1A, Inosine) | 2025 | ModiDeC: a multi-RNA modification classifier for direct nanopore sequencing | <https://github.com/mem3nto0/ModiDeC-RNA-modification-classifier> |
| ***ModQuant*** | Ψ | 2024 | Nanopore signal deviations from pseudouridine modifications in RNA are sequence-specific: quantification requires dedicated synthetic controls | <https://github.com/wanunulab/ModQuant> |
| ***Nanocompore*** | Multiple (m6A, Inosine, m5C, Ψ, m6,2A, m1G) | 2021 | RNA modifications detection by comparative Nanopore direct RNA sequencing | <https://github.com/tleonardi/nanocompore> |
| ***NanoDoc*** | Multiple | 2020 | nanoDoc: RNA modification detection using Nanopore raw reads with Deep One-Class Classification | <https://github.com/uedaLabR/nanoDoc> |
| ***NanoDoc2*** | Multiple | 2023 | RNA Modification Detection Using Nanopore Direct RNA Sequencing and nanoDoc2 | <https://github.com/uedaLabR/nanoDoc2> |
| ***Nanom6A*** | m6A | 2021 | Quantitative profiling of N6-methyladenosine at single-base resolution in stem-differentiating xylem of Populus trichocarpa using Nanopore direct RNA sequencing | <https://github.com/gaoyubang/nanom6A> |
| ***NanoMUD*** | Ψ, m1Ψ | 2024 | NanoMUD: Profiling of pseudouridine and N1-methylpseudouridine using Oxford Nanopore direct RNA sequencing | <https://github.com/ABOMSBI/NanoMUD> |
| ***NanoNm*** | Nm | 2024 | 2′-O-methylation at internal sites on mRNA promotes mRNA stability | <https://github.com/kaifuchenlab/NanoNm> |
| ***NanoPsu*** | Ψ | 2021 | Interferon inducible pseudouridine modification in human mRNA by quantitative nanopore profiling | <https://github.com/sihaohuanguc/Nanopore_psU> |
| ***nanoRMS*** | Ψ, Nm | 2021 | Quantitative profiling of pseudouridylation dynamics in native RNAs with nanopore sequencing | <https://github.com/novoalab/nanoRMS> |
| ***Nm-Nano*** | Nm | 2024 | Nm-Nano: a machine learning framework for transcriptome-wide single-molecule mapping of 2´-O-methylation (Nm) sites in nanopore direct RNA sequencing datasets | <https://github.com/Janga-Lab/Nm-Nano> |
| ***Penguin*** | Ψ | 2022 | Penguin: A tool for predicting pseudouridine sites in direct RNA nanopore sequencing data | <https://github.com/Janga-Lab/Penguin> |
| ***PsiNanopore*** | Ψ | 2023 | Semi-quantitative detection of pseudouridine modifications and type I/II hypermodifications in human mRNAs using direct long-read sequencing | <http://www.github.com/RouhanifardLab/PsiNanopore> |
| ***pum6a*** | m6A | 2025 | Decoding the m6A epitranscriptomic landscape for biotechnological applications using a direct RNA sequencing approach | <https://github.com/liuchuwei/pum6a> |
| ***RedNano*** | m6A | 2024 | RNA m6A detection using raw current signals and basecalling errors from Nanopore direct RNA sequencing reads | <https://github.com/Derryxu/RedNano> |
| ***Remora*** | Multiple | 2021 | ONT Remora | <https://github.com/nanoporetech/remora> |
| ***RNANO*** | Multiple (m6A, m1A, m5C, m7G, ac4C, Nm, Ψ) | 2025 | Accurate prediction of multiple RNA modifications from nanopore direct RNA sequencing data with RNANO | <https://github.com/abhhba999/RNANO> |
| ***SingleMod*** | m6A | 2025 | Single-molecule direct RNA sequencing reveals the shaping of epitranscriptome across multiple species | <https://github.com/xieyy46/SingleMod-v1> |
| ***TandemMod*** | Multiple (m6A, m5C, m7G, Ψ, Inosine) | 2024 | Transfer learning enables identification of multiple types of RNA modifications using nanopore direct RNA sequencing | <https://github.com/yulab2021/TandemMod> |
| ***Tombo*** | Multiple | 2016 | ONT Tombo | <https://github.com/nanoporetech/tombo?utm_source=chatgpt.com> |
| ***xPore*** | m6A | 2021 | Identification of differential RNA modifications from nanopore direct RNA sequencing with xPore | <https://github.com/GoekeLab/xpore> |
| ***Yanocomp*** | m6A | 2021 | Yanocomp: robust prediction of m6A modifications in individual nanopore direct RNA reads | [www.github.com/bartongroup/yanocomp](http://www.github.com/bartongroup/yanocomp) |

**Abbreviations:** Pseudouridine (Ψ), N1-methylpseudouridine (m1Ψ), 2´-O-methylation (Nm)

**Note:** The table only summarizes partial information with utilized models or detailed frameworks; partial omission might occur during the process. In this table, the label “P” denotes presence.

**Supplementary Table 3** m6A sites obtained from different NGS Platforms

| **Technique** | **Total number** | **GEO** | **Reference Paper** | **Reference Paper Link** |
| --- | --- | --- | --- | --- |
| **m6A-seq (25bp)** | 10730 | GSE54365 | Perturbation of m6A writers reveals two distinct classes of mRNA methylation at internal and 5' sites | <https://pubmed.ncbi.nlm.nih.gov/24981863> |
| **PA-m6A-seq** | 17161 | GSE54921 | High-Resolution Mapping of N⁶-Methyladenosine in Transcriptome and Genome Using a Photo-Crosslinking-Assisted Strategy | <https://pubmed.ncbi.nlm.nih.gov/26253971> |
| **m6A-CLIP-seq** | 45349 | GSE71154 | A majority of m6A residues are in the last exons, allowing the potential for 3' UTR regulation | <https://pubmed.ncbi.nlm.nih.gov/26404942> |
|  |  | GSE86336 | m6A mRNA modifications are deposited in nascent pre-mRNA and are not required for splicing but do specify cytoplasmic turnover | <https://pubmed.ncbi.nlm.nih.gov/28637692> |
| **miCLIP** | 64598 | GSE63753 | Single-nucleotide-resolution mapping of m6A and m6Am throughout the transcriptome | <https://pubmed.ncbi.nlm.nih.gov/26121403> |
|  |  | GSE122948 | Identification of the m6Am Methyltransferase PCIF1 Reveals the Location and Functions of m6Am in the Transcriptome | <https://pubmed.ncbi.nlm.nih.gov/31279658> |
|  |  | GSE128699 | The human 18S rRNA m6A methyltransferase METTL5 is stabilized by TRMT112 | <https://pubmed.ncbi.nlm.nih.gov/31328227> |
|  |  | GSE73405 | 5' UTR m(6)A Promotes Cap-Independent Translation | <https://pubmed.ncbi.nlm.nih.gov/26593424> |
|  |  | GSE98623 | The N6-methyladenosine (m6A)-forming enzyme METTL3 controls myeloid differentiation of normal hematopoietic and leukemia cells | <https://pubmed.ncbi.nlm.nih.gov/28920958> |
|  |  | GSE121942 | Histone H3 trimethylation at lysine 36 guides m6A RNA modification co-transcriptionally | <https://pubmed.ncbi.nlm.nih.gov/30867593> |
| **m6ACE-seq** | 1634 | GSE124509 | Atlas of quantitative single-base-resolution N6-methyl-adenine methylomes | <https://www.nature.com/articles/s41467-019-13561-z> |
| **m6A-REF-seq** | 11461 | GSE125240 | Single-base mapping of m6A by an antibody-independent method | <https://pubmed.ncbi.nlm.nih.gov/31281898> |
| **MAZTER-seq** | 14944 | GSE122961 | Deciphering the "m6A Code" via Antibody-Independent Quantitative Profiling | <https://pubmed.ncbi.nlm.nih.gov/31257032> |
| **DART-seq** | 7954 | GSE125780 | DART-seq: an antibody-free method for global m6A detection | <https://pubmed.ncbi.nlm.nih.gov/31548708> |
| **m6A-Label-seq** | 3603 | GSE131316 | A metabolic labeling method detects m6A transcriptome-wide at single base resolution | <https://pubmed.ncbi.nlm.nih.gov/32341503> |

**Supplementary Table 4** Predicted sites and evaluating elements for five m6A detecting tools in HEK293_WT with High / Medium / Low / Very Low confidence levels

| ***m6A*** Model Metrics for ***High Confidence Group*** in HEK293_WT | | | | | | | |
| --- | --- | --- | --- | --- | --- | --- | --- |
| **Tool** | **Predicted m6A Site Numbers** | **TP** | **FP** | **FN** | **Precision** | **Recall** | **F1** |
| **m6Anet** | 36688 | 615 | 36073 | 628 | 0.0168 | 0.4948 | 0.0324 |
| **MINES** | 35870 | 610 | 35260 | 633 | 0.0170 | 0.4907 | 0.0329 |
| **nanom6A** | 30337 | 479 | 29858 | 764 | 0.0158 | 0.3854 | 0.0303 |
| **DRUMMER** | 36 | 12 | 24 | 1231 | 0.3333 | 0.0097 | 0.0188 |
| **DiffErr** | 3 | 2 | 1 | 1241 | 0.6667 | 0.0016 | 0.0032 |
| ***m6A*** Model Metrics for ***Medium Confidence Group*** in HEK293_WT | | | | | | | |
| **Tool** | **Predicted m6A Site Numbers** | **TP** | **FP** | **FN** | **Precision** | **Recall** | **F1** |
| **m6Anet** | 36688 | 2393 | 34295 | 4399 | 0.0652 | 0.3523 | 0.1101 |
| **MINES** | 35870 | 2238 | 33632 | 4554 | 0.0624 | 0.3295 | 0.1049 |
| **nanom6A** | 30337 | 1730 | 28607 | 5062 | 0.0570 | 0.2547 | 0.0932 |
| **DRUMMER** | 36 | 13 | 23 | 6779 | 0.3611 | 0.0019 | 0.0038 |
| **DiffErr** | 3 | 0 | 3 | 6792 | 0 | 0 | NA |
| ***m6A*** Model Metrics for ***Low Confidence Group*** in HEK293_WT | | | | | | | |
| **Tool** | **Predicted m6A Site Numbers** | **TP** | **FP** | **FN** | **Precision** | **Recall** | **F1** |
| **m6Anet** | 36688 | 5686 | 31002 | 20609 | 0.1550 | 0.2162 | 0.1806 |
| **MINES** | 35870 | 5154 | 30716 | 21141 | 0.1437 | 0.1960 | 0.1658 |
| **nanom6A** | 30337 | 3950 | 26387 | 22345 | 0.1302 | 0.1502 | 0.1395 |
| **DRUMMER** | 36 | 5 | 31 | 26290 | 0.1389 | 0.0002 | 0.0004 |
| **DiffErr** | 3 | 0 | 3 | 26295 | 0 | 0 | NA |
| ***m6A*** Model Metrics for ***VeryLow Confidence Group*** in HEK293_WT | | | | | | | |
| **Tool** | **Predicted m6A Site Numbers** | **TP** | **FP** | **FN** | **Precision** | **Recall** | **F1** |
| **m6Anet** | 36688 | 8294 | 28394 | 91037 | 0.2261 | 0.0835 | 0.1220 |
| **MINES** | 35870 | 6790 | 29080 | 92541 | 0.1893 | 0.0684 | 0.1004 |
| **nanom6A** | 30337 | 6613 | 23724 | 92718 | 0.2180 | 0.0666 | 0.1020 |
| **DRUMMER** | 36 | 2 | 34 | 99329 | 0.0556 | 0 | 0 |
| **DiffErr** | 3 | 0 | 3 | 99331 | 0 | 0 | NA |

**Supplementary Table 5** Predicted sites and evaluating elements for five m6A detecting tools in HMEC_WT with High / Medium / Low / Very Low confidence levels

| ***m6A*** Model Metrics for ***High Confidence Group*** in HMEC_WT | | | | | | | |
| --- | --- | --- | --- | --- | --- | --- | --- |
| **Model** | **Predicted** | **TP** | **FP** | **FN** | **Precision** | **Recall** | **F1** |
| **m6Anet** | 26143 | 499 | 25644 | 744 | 0.0191 | 0.4014 | 0.0364 |
| **nanom6A** | 23639 | 390 | 23249 | 853 | 0.0165 | 0.3138 | 0.0313 |
| **MINES** | 13006 | 368 | 12638 | 875 | 0.0283 | 0.2961 | 0.0517 |
| **DRUMMER** | 23 | 3 | 20 | 1240 | 0.1304 | 0.0024 | 0.0047 |
| **DiffErr** | 19 | 0 | 19 | 1243 | 0 | 0 | NA |
| ***m6A*** Model Metrics for ***Medium Confidence Group*** in HMEC_WT | | | | | | | |
| **Model** | **Predicted** | **TP** | **FP** | **FN** | **Precision** | **Recall** | **F1** |
| **m6Anet** | 26143 | 1767 | 24376 | 5025 | 0.0676 | 0.2602 | 0.1073 |
| **nanom6A** | 23639 | 1282 | 22357 | 5510 | 0.0542 | 0.1888 | 0.0843 |
| **MINES** | 13006 | 1119 | 11887 | 5673 | 0.0860 | 0.1648 | 0.1130 |
| **DRUMMER** | 23 | 5 | 18 | 6787 | 0.2174 | 0.0007 | 0 |
| **DiffErr** | 19 | 1 | 18 | 6791 | 0.0526 | 0.0001 | 0.0003 |
| ***m6A*** Model Metrics for ***Low Confidence Group*** in HMEC_WT | | | | | | | |
| **Model** | **Predicted** | **TP** | **FP** | **FN** | **Precision** | **Recall** | **F1** |
| **m6Anet** | 26143 | 3857 | 22286 | 22438 | 0.1475 | 0.1467 | 0.1471 |
| **nanom6A** | 23639 | 2949 | 20690 | 23346 | 0.1248 | 0.1122 | 0.1181 |
| **MINES** | 13006 | 2265 | 10741 | 24030 | 0.1742 | 0.0861 | 0.1153 |
| **DRUMMER** | 23 | 8 | 15 | 26287 | 0.3478 | 0.0003 | 0.0006 |
| **DiffErr** | 19 | 2 | 17 | 26293 | 0.1053 | 0.0001 | 0.0002 |
| ***m6A*** Model Metrics for ***VeryLow Confidence Group*** in HMEC_WT | | | | | | | |
| **Model** | **Predicted** | **TP** | **FP** | **FN** | **Precision** | **Recall** | **F1** |
| **m6Anet** | 26143 | 5702 | 20441 | 93629 | 0.2181 | 0.0574 | 0.0909 |
| **nanom6A** | 23639 | 5101 | 18538 | 94230 | 0.2158 | 0.0514 | 0.083 |
| **MINES** | 13006 | 2377 | 10629 | 96954 | 0.1828 | 0.0239 | 0.0423 |
| **DRUMMER** | 23 | 3 | 20 | 99328 | 0.1304 | 0 | 0.0001 |
| **DiffErr** | 19 | 5 | 14 | 99326 | 0.2632 | 0.0001 | 0.0001 |

**Supplementary Table 6** Predicted sites and evaluating elements for three PseudoU detecting tools in HEK293_WT and HMEC_WT

| ***PseudoU*** Detection Model Metrics in HEK293_WT | | | | | | | |
| --- | --- | --- | --- | --- | --- | --- | --- |
| **Model** | **Predicted** | **TP** | **FP** | **FN** | **Precision** | **Recall** | **F1** |
| **Nanomud** | 82581 | 57 | 82524 | 5648 | 0.0007 | 0.01 | 0.0013 |
| **Nanopsu** | 10979 | 31 | 10948 | 5674 | 0.0028 | 0.0054 | 0.0037 |
| **Penguin** | 13661 | 0 | 13661 | 5705 | 0 | 0 | NA |
| ***PseudoU*** Detection Model Metrics in HMEC_WT | | | | | | | |
| **Model** | **Predicted** | **TP** | **FP** | **FN** | **Precision** | **Recall** | **F1** |
| **Nanomud** | 32139 | 33 | 32106 | 5672 | 0.001 | 0.0058 | 0.0017 |
| **Nanopsu** | 4579 | 16 | 4563 | 5689 | 0.0035 | 0.0028 | 0.0031 |
| **Penguin** | 644248 | 74 | 644174 | 5631 | 0.0001 | 0.013 | 0.0002 |

***Figure: upload separately as Supplementary Figure1_newadd.pdf***

**Supplementary Figure 1** Additional information about m6A Detection using 5 different tools of ***m6Anet***, ***MINES***, ***nanom6A***, ***DRUMMER*** and ***DiffErr*** in HEK293_WT and HMEC_WT. **(A)** Percentages of Overlapped and Non-overlapped sites with reference NGS datasets for five tools across four confidence groups. **(B)** Percentage of Four differnce confidence groups in reference NGS datasets. **(C)** Precision and Recall measures of five tools across four confidence groups for both HEK293_WT and HMEC_WT.
